# Harnessing probiotics against candidiasis: a systematic review of experimental and mechanistic evidence

**DOI:** 10.3389/fcimb.2026.1894309

**Published:** 2026-09-24

**Authors:** Taparia Saloni, Nagaraju Chaithra, Shivaswamy Umamaheshwari

**Affiliations:** 1 Department of Microbiology, School of Life Sciences, JSS Academy of Higher Education & Research, Mysuru, India; 2 Division of Medical Statistics, School of Life Sciences, JSS Academy of Higher Education & Research, Mysuru, India

**Keywords:** anti-*Candida* activity, biofilm, *Candida* species, candidiasis, microbiome, postbiotics, probiotics, systematic review

## Abstract

**Background:**

*Candida* species are among the most important opportunistic fungal pathogens responsible for superficial mucosal infections and life-threatening invasive candidiasis, particularly in immunocompromised individuals. The increasing prevalence of antifungal resistance, biofilm-associated tolerance, and the emergence of multidrug-resistant species such as *Candida auris* have highlighted the need for alternative or adjunctive therapeutic strategies. Probiotics have emerged as promising microbiome-based interventions owing to their ability to inhibit *Candida* growth, suppress virulence traits, restore microbial homeostasis, and modulate host immune responses.

**Methods:**

This systematic review comprehensively evaluated the experimental and mechanistic evidence regarding the anti-*Candida* activity of probiotics and probiotic-derived products. A systematic literature search was conducted in PubMed, Scopus, and Web of Science for studies published between 1 January 2015 and 5 January 2026 in accordance with PRISMA 2020 guidelines. Studies meeting the predefined eligibility criteria were included in the qualitative synthesis.

**Results:**

Eighteen studies met the predefined eligibility criteria. Most studies investigated *Lactobacillus* species, followed by *Bifidobacterium*, *Saccharomyces*, and probiotic-derived metabolites or postbiotics, using predominantly *in vitro* models with limited *in vivo* evidence. Across studies, probiotics consistently demonstrated anti-*Candida* activity through inhibition of fungal growth, adhesion, yeast-to-hyphal transition, biofilm formation, and virulence-associated gene expression. Additional mechanisms included the production of antimicrobial metabolites, competitive exclusion, immune modulation, enhancement of epithelial barrier function, and restoration of microbial homeostasis. Emerging evidence also supports the antifungal potential of postbiotics and activity against non-albicans *Candida* species and *Candida auris*, although available evidence remains limited. Considerable heterogeneity in probiotic strains, experimental models, methodologies, and outcome measures precluded quantitative meta-analysis.

**Conclusions:**

Current evidence supports probiotics and postbiotics as promising microbiome-based strategies for reducing *Candida* pathogenicity; however, the available evidence remains predominantly preclinical. Well-designed clinical studies are required to establish strain-specific efficacy, optimal formulations, dosing strategies, safety, and therapeutic applicability before routine clinical implementation.

**Systematic review registration:**

https://www.crd.york.ac.uk/prospero/view/CRD420261278363, identifier CRD420261278363.

## 1 Introduction

*Candida* species represent a major group of opportunistic fungal pathogens associated with a broad spectrum of clinical manifestations, ranging from superficial mucocutaneous infections to life-threatening invasive candidiasis, with increased prevalence, morbidity and mortality among immunocompromised individuals (Rossoni et al., 2018a). The global burden of candidiasis continues to increase owing to widespread use of invasive medical procedures, broad-spectrum antibiotics, immunosuppressive therapies, and the expanding population of susceptible hosts. Invasive candidiasis represents one of the most common healthcare-associated fungal infections worldwide and is associated with mortality rates ranging from 30% to 60%, depending on the patient population and healthcare setting (Bongomin et al., 2017). Looking into this growing threat, the World Health Organization (WHO) has introduced the Fungal Priority Pathogens List (FPPL), which categorizes them into critical, high, and medium priority groups, with *Candida* species represented across all categories, highlighting their global clinical and public health significance (World Health Organization, 2022).

Under normal conditions, *Candida* species exist as commensal yeasts colonizing mucosal and epithelial surfaces, including the oral cavity, gastrointestinal tract, urogenital tract, skin, and respiratory tract (Kim and Kang, 2019; Mundula et al., 2019). However, under predisposing conditions such as immunosuppression, malignancy, diabetes mellitus, HIV/AIDS, prolonged antibiotic exposure, and disruption of the host microbiota they undergo a phenotypic transition from commensalism to pathogenicity through a yeast-to-hyphal morphological switch (Wang et al., 2017; Kim and Kang, 2019). This transition facilitates tissue invasion, immune evasion, and biofilm formation, thereby contributing to increased pathogenicity and the development of both localized and systemic infections.

The emergence of non-*albicans Candida* (NAC), including *Candida tropicalis, Candida glabrata, Candida parapsilosis, Candida krusei and Candida lusitaniae* has further complicated clinical management due to their intrinsic and acquired antifungal resistance profiles (Bongomin et al., 2017; Andrade et al., 2022). In addition, *Candida auris* has emerged as a multidrug-resistant pathogen associated with healthcare-associated outbreaks, high transmissibility, and significant mortality, posing a significant global health challenge.

A key determinant of *Candida* pathogenicity is its ability to form biofilms on mucosal surfaces and medical devices. These structured microbial communities are embedded in an extracellular matrix that limits antifungal penetration and enhances resistance to host defenses, contributing to persistent and recurrent infections. Disruption of normal microbiota further exacerbates disease progression by reducing colonization resistance and impairing immune homeostasis.

The increasing prevalence of antifungal resistance has further complicated candidiasis management. The prevalence of azole resistance has increased greatly recently in NAC and in *Candida auris*. This has reduced the effectiveness of commonly employed antifungals, such as fluconazole. Echinocandins, which form the basis for treatment of invasive candidiasis, have also shown increasing resistance too, especially when dealing with *C. glabrata* infections. Another important factor contributing to antifungal failure is biofilm tolerance. It is characterized by antifungal tolerance shown by sessile cells of *Candida* present in biofilms.

Although antifungal agents remain the mainstay of therapy, their clinical effectiveness is increasingly limited by toxicity, emerging resistance, and poor activity against biofilm-associated cells. These challenges underscore the urgent need for alternative or adjunctive therapeutic strategies that can target *Candida* virulence while restoring microbial homeostasis.

Recent advances in microbiome research have highlighted the critical role of commensal microorganisms in maintaining mucosal homeostasis, providing colonization resistance against opportunistic pathogens and regulating the host immune responses. Disruption of the host microbiota has been recognized as an important predisposing factor for *Candida* overgrowth and infection. Consequently, probiotic-based interventions have attracted increasing attention as potential microbiome-based strategies capable of inhibiting *Candida* growth, suppressing virulence-associated traits, restoring microbial balance, and enhancing host defense mechanisms. Unlike conventional antifungal agents, probiotics may exert their effects through multiple complementary mechanisms, including direct inhibition of *Candida* growth, suppression of virulence traits, restoration of microbial homeostasis, and modulation of host immune responses (D’Enfert et al., 2021; Li et al., 2022).

In this context, probiotics have emerged as potential biotherapeutic agents with antifungal, antibiofilm, and immunomodulatory properties (Jørgensen et al., 2017; Hu et al., 2019; Carvalho et al., 2021). Defined as live microorganisms that confer health benefits to the host when administered in adequate amounts, probiotics, particularly species belonging to *Lactobacillus, Bifidobacterium, and Saccharomyces* have demonstrated inhibitory effects against *Candida* through multiple mechanisms. These include competitive exclusion, production of antimicrobial metabolites, inhibition of adhesion and biofilm formation, enhancement of epithelial barrier function, and modulation of host immune responses (Zhao et al., 2016).

Although multiple studies have evaluated the effectiveness of probiotics for their anti-*Candida* activity, the available evidence remains fragmented due to substantial heterogeneity in probiotic strains, experimental models, methodologies and outcome measures. Furthermore, there has been considerable research on *C. albicans* in the past; however, Comparatively relatively few investigations into NAC species, newly emerging species like *C. auris* mechanisms related to pathogenicity (such as biofilm production and phenotypic switching), and even the use of metabolites and postbiotics from probiotics. To our knowledge, no previous systematic review has comprehensively integrated the experimental and mechanistic evidence regarding probiotic-mediated anti-*Candida* activity across diverse *Candida* species, virulence mechanisms, and probiotic-derived products.

Therefore, this systematic review aims to comprehensively evaluate mechanistic and experimental evidence on the role of probiotics in the prevention and management of candidiasis, with emphasis on anti-*Candida* activity, biofilm inhibition, virulence suppression, and host immune modulation.

## 2 Methods

### 2.1 Protocol and study registration

This systematic review was conducted in accordance with the Preferred Reporting Items for Systematic Reviews and Meta-Analyses (PRISMA 2020) guidelines. The review protocol was prospectively registered in the International Prospective Register of Systematic Reviews (PROSPERO) under registration number CRD420261278363 before completion of data extraction and analysis. A predefined methodological framework was established before study selection to ensure transparency, reproducibility and methodological rigor throughout the review process while minimizing the risk of selection and reporting bias at each stage.

### 2.2 Eligibility criteria

A thorough literature search was done to identify relevant studies published between 1 January 2015 and 5 January 2026 representing the most recent 10-year evidence base available on probiotic interventions against *Candida* species. Eligibility was restricted to peer-reviewed original research articles published in English, owing to practical considerations related to language accessibility during screening and data extraction.

Studies were considered eligible if they investigated the anti-*Candida*, antibiofilm, anti-virulence or immunomodulatory effects of probiotics, probiotic-derived metabolites against *Candida* species using experimental *in vitro* and/or *in vivo* models or clinical study designs.

Eligible studies were required to report at least one predefined antifungal-related outcome, including inhibition of fungal growth, adhesion, germ tube formation, yeast-to-hyphal transition, virulence factors, biofilm development or modulation of host immune responses. Studies were excluded if they were review articles, editorials, book chapters, conference abstracts, or case reports. Additionally, studies not involving probiotics as an intervention, studies with incomplete or irrelevant data, non-English publications, and studies published before 1 January 2015 were excluded from the review. A summary of the predefined inclusion and exclusion criteria is presented in **Table 1**.

**Table 1:** Summary of Eligibility criteria used for study selection.

Category	Inclusion criteria	Exclusion criteria
Publication type	Peer-reviewed original research articles	Review articles, editorials, book chapters, conferences abstracts, case reports
Language	English-language publications	Non-English publications
Search period	Studies published between 1 January 2015 and 5 January 2026	Studies published before 1 January 2015 and after 5 January 2026
Target organism	*Candida albicans, non-albicans Candida and Candida auris*	Studies not involving *Candida* species.
Intervention	Probiotics, probiotic-derived metabolites	Studies not involving probiotic-based interventions
Outcomes	Anti-*Candida* activity, biofilm inhibition, virulence suppression, immunomodulatory effects	Studies reporting no relevant anti-Candida outcomes.
Data availability	Studies reporting sufficient methodological and outcome data	Studies with irrelevant, incomplete or insufficient data

Although clinical studies were considered eligible during screening, no clinical studies meeting the predefined inclusion criteria were ultimately identified for inclusion in the final qualitative synthesis.

A quantitative meta-analysis could not be performed due to high heterogeneity among the included studies. The heterogeneity arose from differences among the studies due to the use of different probiotic strains such as *Lactobacillus paracasei, Lactobacillus rhamnosus, Lactobacillus fermentum, Lactobacillus acidophilus, Bacillus subtilis*., etc. differences in the use of *Candida* species such as *Candida albicans, Candida krusei, Candida tropicalis, Candida glabrata., etc*, experimental models (*in vitro*, *in vivo*), different in the dosages and treatment durations, methods adapted for evaluating the anti-*Candida* activity, and outcomes measures. These differences limited the feasibility of performing a meta-analysis.

### 2.3 Search strategy

A systematic literature search of PubMed, Web of Science, and Scopus was conducted to identify relevant studies published between 1 January 2015 and 5 January 2026, with the final search completed on 5 January 2026. These databases were selected because they provide comprehensive coverage of biomedical, microbiological and interdisciplinary scientific literature, thereby maximizing the identification of relevant studies investigating probiotic interventions against *Candida* species.

The search strategy was developed through preliminary scoping searches, including Medical Subject Headings (MeSH) terminology and examination of keywords used in previously published literature. Both controlled vocabulary terms and free-text keywords were combined using Boolean operators (AND, OR, and NOT) to ensure comprehensive yet targeted retrieval of relevant studies.

A broad search strategy combining probiotic-related and *Candida-*related terms was used to maximize sensitivity and minimize the risk of missing potentially relevant studies. A highly sensitive search strategy was adopted because probiotic-*Candida* research encompasses diverse probiotic strains, *Candida* species, probiotic derived products and outcome measures. Although this results in a large number of records, it ensured comprehensive retrieval of all the available evidence and minimizes the risk of excluding potentially relevant studies. Outcome-specific keywords such as antibiofilm activity, virulence inhibition, and immunomodulation were intentionally not included to avoid introducing selection bias. The core search string used was:

(“probiotic” OR “Lactobacillus” OR “Bifidobacterium” OR “Saccharomyces”) AND (“Candida” OR “candidiasis”).

The complete database-specific search strategies are provided in **Supplementary Table 1**.

### 2.4 PICO framework

The review was structured according to the PICO framework:

- Population (P): Experimental *in vitro* and *in vivo* models involving *Candida* species, including *C. albicans*, NAC species, and *C. auris*.
- Intervention (I): Administration of probiotic strains, probiotic-derived metabolites, or formulations via any route.
- Comparison (C): Untreated controls or standard antifungal therapies.
- Outcomes (O): Antifungal activity, reduction in fungal growth or colony-forming units (CFU), inhibition of adhesion and biofilm formation, suppression of virulence factors and yeast-to-hyphal transition, modulation of host immune responses, and restoration of microbial balance.

### 2.5 Literature screening process

All retrieved records were imported into Microsoft Excel for organization and management. Duplicate records were identified and removed prior to screening. A two-stage screening process was conducted independently, beginning with title and abstract screening to exclude irrelevant studies, followed by full-text assessment based on predefined eligibility criteria.

Disagreements between reviewers during screening and eligibility assessment were resolved through discussion and consensus. Where necessary, a third reviewer was consulted to resolve discrepancies. Reasons for exclusion at the full-text stage were documented and incorporated into the PRISMA 2020 flow diagram.

### 2.6 Data extraction

Data were collected using a predefined extraction sheet created in Microsoft Excel before the review commenced. Two reviewers (ST and CN) independently extracted the relevant study information. Any disagreements identified during the extraction process were discussed and resolved with the involvement of a third reviewer (US).

The following information was extracted from each study:

- first author and year of publication
- country of origin,
- study design,
- probiotic strain(s) or postbiotic intervention,
- target *Candida* species,
- experimental model and methodology,
- Key Outcomes,

### 2.7 Quality assessment

Methodological quality assessment of the included studies was conducted using a predefined methodological appraisal checklist developed by the authors and adapted from previously reported quality assessment approaches used for experimental *in vitro* and *in vivo* studies. Since no validated appraisal tool exists for heterogeneous mechanistic probiotic-*Candida* studies, the checklist was developed by adapting methodological domains commonly reported in previous systematic reviews.

The appraisal checklist evaluated eight methodological domains:

1. clarity of study objective
2. appropriateness of study design,
3. identification of probiotic strain or intervention,
4. identification of *Candida* species,
5. adequacy and reproducibility of experimental methods,
6. clarity of outcome reporting,
7. inclusion of appropriate control or comparator groups.
8. quantitative outcome assessment and statistical reporting,

Each item was scored as 1 if the information was clearly provided and 0 if no information, vague description, or insufficient detail was provided. Hence, the maximum number of scores was 8 points per study.

Based on total score, studies were classified as:

- 0-2 = low quality
- 3-5 = moderate quality
- 6-8 = high quality

Quality assessment was performed independently by two reviewers, and any disagreements were resolved through discussion. The complete methodological appraisal criteria and the quality results of included studies are summarized in **Table 2**. The use of a tailored methodological appraisal checklist was adapted as it enabled evaluation of study-specific methodological features relevant to experimental probiotic-*Candida* research, including probiotic characterization, *Candida* species identification and mechanistic outcome measurement which are not completely addressed by conventional clinical risk-of-bias tools. However, this approach has certain limitations as the use of a customised appraisal framework may limit the direct comparison with quality assessments reported in other systematic reviews employing standardized assessment tools.

**Table 2:** Methodological quality assessment of studies included in the systematic review.

Study	Clear objective	Study design	Probiotic intervention reported	*Candida* species reported	Methods clearly described	Outcomes clearly reported	Control/Comparator reported	Quantitative outcome assessment & statistical reporting	Total Score (/8)	Overall Quality
(Chantanawilas et al., 2024)	1	1	1	1	1	1	1	1	**8**	High
(Hashem and Hashem, 2021)	1	1	1	1	1	1	1	0	**7**	High
(De Gregorio et al., 2019)	1	1	1	1	1	1	1	1	**8**	High
(Gharieb et al., 2024)	1	1	1	1	1	1	1	0	**7**	High
(Rossoni et al., 2018a)	1	1	1	1	1	1	1	1	**8**	High
(Salari and Ghasemi Nejad Almani, 2020)	1	1	1	1	1	1	1	0	**7**	High
(Wang et al., 2017)	1	1	1	1	1	1	1	1	**8**	High
(Ardestani et al., 2022)	1	1	1	1	1	1	1	0	**7**	High
(Zeng et al., 2023)	1	1	1	1	1	1	1	1	**8**	High
(Inturri et al., 2019)	1	1	1	1	1	1	1	0	**7**	High
(Yocheva et al., 2024)	1	1	1	1	1	1	1	0	**7**	High
(Poon and Hui, 2023)	1	1	1	1	1	1	1	1	**8**	High
(Takano et al., 2023)	1	1	1	1	1	1	0	1	**7**	High
(do Carmo et al., 2016)	1	1	1	1	1	1	1	0	**7**	High
(Buonanno et al., 2025)	1	1	1	1	1	1	1	1	**8**	High
(de Barros et al., 2018)	1	1	1	1	1	1	1	1	**8**	High
(Matsubara et al., 2016)	1	1	1	1	1	1	1	0	**7**	High
(Rossoni et al., 2020)	1	1	1	1	1	1	1	1	**8**	High

Each methodological domain was scored as 1 (adequately reported) or 0 (unclear/not reported). Maximum possible score = 8. Studies scoring 6 -8 were classified as high quality, 3 -5 as moderate quality, and 0 -2 as low quality. Bold values indicate the total methodological quality score for each study.

### 2.8 Risk of bias assessment

Risk of bias assessment was assessed using a domain-based methodological quality assessment approach adapted from Cochrane risk-of-bias principles to evaluate the included *in vitro* and *in vivo* studies. Four domains were evaluated: selection bias, performance bias, detection bias, and reporting bias. Selection bias was assessed based on the adequacy of experimental group selection and the identification of probiotic interventions and *Candida* species. Performance bias evaluated the experimental procedures, treatment conditions and intervention protocols. Detection bias assessed the objectivity, reproducibility and appropriateness of outcome measurements. Reporting bias evaluated the transparency and consistency of outcomes reported. Each domain was categorized as low risk, some concerns, or high risk according to the methodology adopted and the outcomes reported. A complete risk of bias judgment was determined for each study using a conservative approach, whereby the final rating reflected the highest level of concern identified across the assessed domains. As the included evidence primarily consisted of experimental laboratory studies rather than randomized clinical trials, the assessment framework was modified to better align with the methodological characteristics of probiotic research. Standardized risk-of-bias tools such as SYRCLE and Joanna Briggs Institute (JBI) checklists were not fully applicable due to the heterogeneous nature of *in vitro* and *in vivo* experimental designs. Therefore, a modified domain-based assessment framework was employed to better capture the methodological characteristics of probiotic-*Candida* experimental studies.

For graphical representation, the risk-of-bias assessments were presented using the robvis tool, which was employed to generate traffic-light and summary bar plots for better interpretation of methodological quality across the included studies.

## 3 Results

### 3.1 Study selection

A total of 36,955 records were identified through database searching, including Scopus (29,992), Web of Science (6,218), and PubMed (745). Prior to screening, 11,703 records were removed as non-research articles and 2,519 duplicates were excluded, leaving 22,733 records for title and abstract screening.

During title and abstract screening, 22,609 records were excluded because they were unrelated to the review objective or did not meet the predefined eligibility criteria. A total of 124 full-text articles were subsequently assessed for eligibility.

Following full-text review, 106 articles were excluded due to insufficient methodological detail, absence of probiotic-based interventions, or failure to report predefined anti-*Candida* outcomes, inappropriate study designs or failure to meet predefined inclusion criteria. The substantial reduction from 36,955 initially identified records to 18 included studies reflects the use of a highly sensitive search strategy designed to maximize the retrieval of potentially relevant literature. Most records were excluded during title and abstract screening because they were unrelated to probiotic-mediated *anti-Candida* activity, whereas full-text exclusions primarily resulted from the absence of probiotic interventions, failure to report predefined anti-*Candida* outcomes, insufficient methodological detail, or failure to satisfy the predefined eligibility criteria. Finally, 18 studies met the predefined inclusion criteria and were included in the qualitative synthesis. No eligible clinical studies meeting the predefined inclusion criteria were identified.

The study selection process involved a sequential screening of records identified through all the databases followed by removal of duplicates. Later it was screened based on title and abstracts for the relevance of study including a full-text evaluation as per the predefined eligibility criteria. The complete study selection process is illustrated in the PRISMA flow diagram (**Figure 1**).

**Figure 1:**
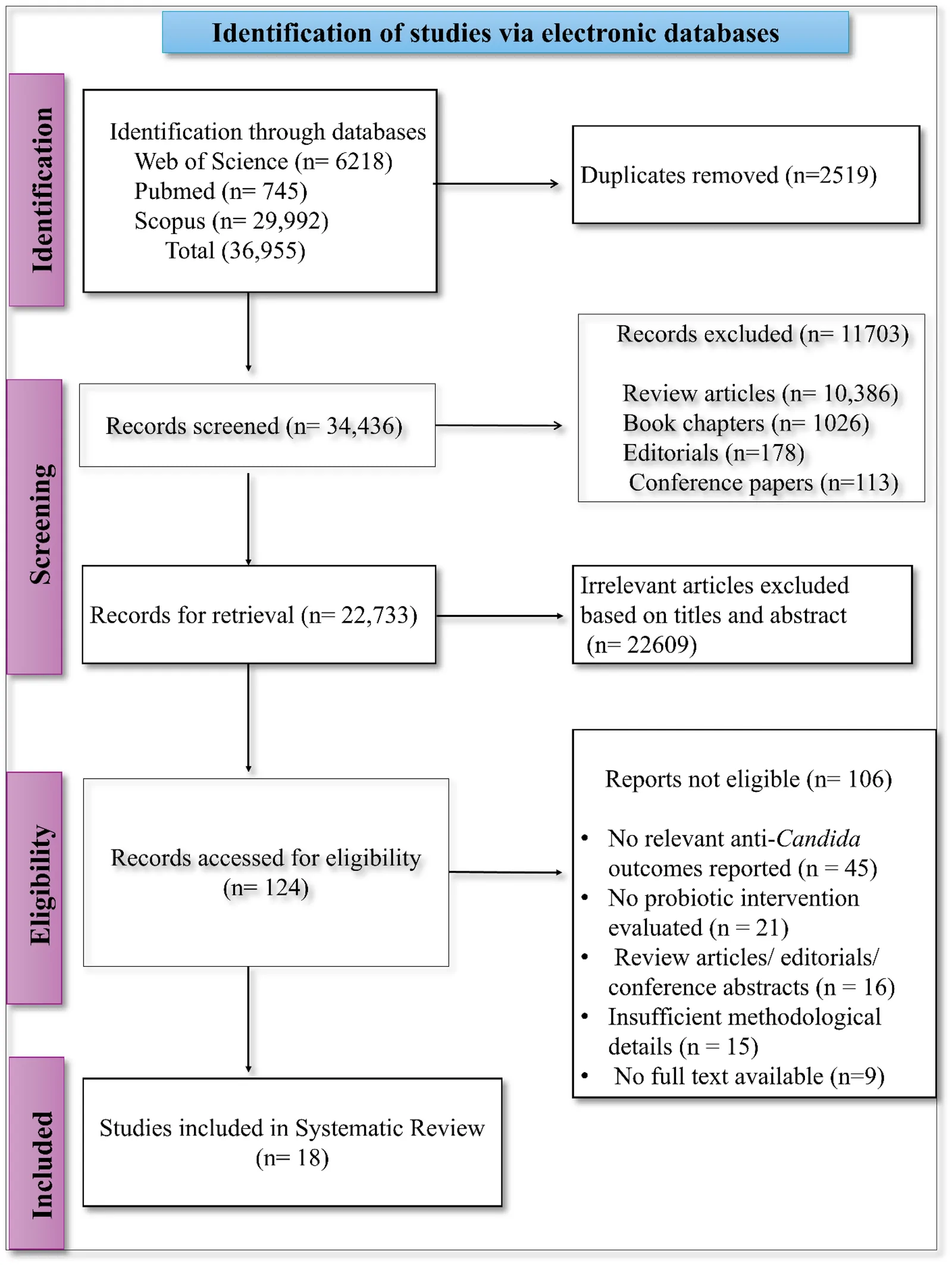
Prisma flow diagram.

The geographical distribution of included studies is presented in **Supplementary Figure 1**. Brazil contributed the highest number of studies (n=5), followed by China, Egypt, and Iran, while the remaining studies originated from Thailand, Italy, Bulgaria, Japan, the United States, and Argentina.

### 3.2 Characteristics of included studies

The characteristics of the included studies are summarized in **Table 3**. The 18 included studies were published between 2015 and 2026 and were conducted across diverse geographical regions, including Thailand, China, Brazil, Iran, India, Egypt, Malaysia, Italy, Bulgaria, and Japan,

**Table 3:** Characteristics of studies evaluating probiotic and postbiotic activity against *Candida* species.

Author, year	Study type	Study country	Experimental model	Probiotic/Postbiotic intervention	Target *Candida* spp.	Main assays	Mechanism of action	Key findings	Limitations
(Chantanawilas et al., 2024)	*In vitro*	Thailand	*human oral keratinocyte cell line H357;*	*L. paracasei*, *L. rhamnosus*, *L. fermentum* strains and CFS	*C. albicans*, *C. dubliniensis*, *C. glabrata*, *C. krusei*, *C. tropicalis*	Aggregation, adhesion inhibition, epithelial adherence assays	Competitive exclusion through co-aggregation; competition for epithelial adhesion sites; effects of surface hydrophobicity and charge; anti-adhesive activity of CFS metabolites	Probiotic strains and CFS significantly reduced *Candida* adhesion; *L. fermentum* SD7 and *L. rhamnosus* SD4 showed strongest anti-adhesion effects.	Cell-line model may not fully reflect *in vivo* mucosa; some assays used reference strains only; active CFS compounds not identified; no animal or clinical validation
(Hashem and Hashem, 2021)	*In vitro*	Egypt	*Clinical oral Candida isolates*	*L. acidophilus* and *L. plantarum* CFS	*C. albicans*, *C. tropicalis*, *C. krusei*	Antifungal susceptibility, adhesion assay, Biofilm assay, germ tube inhibition	*Antifungal metabolite production, inhibition of adhesion and germ tube formation*	*Candida* isolates exhibited resistance to fluconazole, ketoconazole, and amphotericin B. CFS inhibited biofilm formation and germ tube development; *L. acidophilus* showed stronger antibiofilm activity.	*Small isolate number and lack of animal validation.*
(De Gregorio et al., 2019)	*In vitro* and *in vivo*	Argentina	*murine intravaginal candidiasis model*	*Lactobacillus* strains and CFS	*C. albicans*, *C. krusei*, *C. tropicalis*	Anti-*Candida* assays, H_2_O_2_ production, murine model	organic acid production lowering vaginal pH; competitive exclusion on vaginal mucosa; reduction of local inflammation in combined preventive-therapeutic regimen	Strain-dependent antifungal activity observed; *L. rhamnosus* CRL1332 reduced vaginal candidiasis *in vivo*.	Murine model may not fully translate to humans; only two strains tested *in vivo*; effect mainly pH/organic acid mediated; no clinical trial
(Gharieb et al., 2024)	*In vitro*	Egypt	*Wild-type Bacillus subtilis NAM soil isolate tested against clinical C. albicans isolates; CFS and crude extract evaluated*	*Bacillus subtilis* NAM CFS	*C. albicans*	Anti-*Candida* assay, Germ tube inhibition, TEM, GC–MS	Antifungal lipopeptides and enzymes in CFS; enzymatic cell wall degradation; biofilm disruption; fungicidal action at higher concentrations	CFS inhibited growth and germ tube formation and induced ultrastructural damage in *C. albicans*.	*In vitro* only; single *Bacillus* strain; bioactive compounds only partially characterized; no *in vivo* safety/efficacy data; molecular mechanism incompletely defined
(Rossoni et al., 2018a)	*In vitro*	Brazil	*Lactobacillus strains isolated from saliva of 41 caries-free volunteers*	*L. paracasei*, *L. rhamnosus*, *L. fermentum*	*C. albicans*	Biofilm assay, SEM, gene expression	Downregulation of virulence and biofilm-related genes; inhibition of biofilm formation; retardation of hyphal morphogenesis; competitive exclusion	Reduced biofilm formation, hyphal development through downregulation of virulence-associated gene.	*In-vitro* study and only the use of *Candida albicans.*
(Salari and Ghasemi Nejad Almani, 2020)	*In vitro*	Iran	*Oral Candida isolates from patients*	*L. acidophilus* and *L. plantarum*, CFS	*C. albicans*, *C. parapsilosis*, *C. glabrata*, *C. kefyr*, *C. krusei*	MIC/MFC, co-aggregation assays	Growth inhibition, co-aggregation, antibiofilm activity	Demonstrated strong antifungal and co-aggregation activity; comparable or higher activity than fluconazole in some isolates.	Limited to *in vitro* experiments; no molecular mechanistic studies.
(Wang et al., 2017)	*In vitro*	China	*In vitro hyphal induction model*	*L. crispatus*, *L. gasseri*, *L. jensenii*,CFS	*C. albicans*	Antimicrobial activity of *Lactobacilli* Hyphal inhibition, qRT-PCR	*Suppression of yeast-to-hyphal transition and virulence gene expression*	*L. crispatus* showed strongest inhibition of growth and yeast-to-hyphal transition via downregulation of virulence genes.	*Only C. albicans was investigated; no in vivo validation.*
(Ardestani et al., 2022)	*In vitro*	Iran	*In vitro oral candidiasis model*	*Saccharomyces boulardii* extract	*Candida albicans*	Biofilm assay	Biofilm inhibition, reduction of fungal viability	Dose-dependent inhibition of biofilm biomass and viability.	Only evaluated biofilm inhibition; no mechanistic or *in vivo* studies.
(Zeng et al., 2023)	*In vitro*	USA	*In vitro biofilm model*	*L. plantarum* and *L. salivarius*	*C. albicans*	Biofilm model, gene expression	Biofilm disruption and virulence gene suppression	Reduced biofilm biomass and suppressed virulence-associated genes.	Findings were restricted to *in vitro* conditions without host interaction studies.
(Inturri et al., 2019)	*In vitro*	Italy	*In vitro antimicrobial susceptibility model*	*Bifidobacterium longum* BB536 and *L. rhamnosus* HN001 CFS	*C. albicans, C. glabrata, C. tropicalis, C. krusei, C. parapsilosis*	Agar diffusion, MIC assays	Organic acid production, bacteriocin-like peptides, metabolite-mediated antifungal activity	Synergistic antifungal activity mediated by organic acids and peptides.	No *in vivo* validation; mechanisms not completely characterized.
(Yocheva et al., 2024)	*In vitro*	Bulgaria	*In vitro* adhesion model	Multiple *Lactobacillus* strains	*C. albicans*	Co-aggregation, diffusion assays	Fungistatic activity and competitive exclusion	Strong fungistatic and co-aggregation activity against *C. albicans*.	Molecular mechanisms underlying antifungal activity were not investigated
(Poon and Hui, 2023)	*In vitro*	China	Multispecies planktonic and biofilm model with *Streptococcus mutans*	*L. rhamnosus*, *L. plantarum*, *L. acidophilus* CFS	*C. albicans*, *C. tropicalis*, *C. parapsilosis*	Growth inhibition assay, hyphal inhibition assay, Biofilm assay, Filamentation assay qRT-PCR and gene expression	Growth inhibition, suppression of yeast-to-hyphal transition, biofilm inhibition	*L. plantarum* significantly inhibited *C. albicans* growth (approximately 2-log reduction), suppressed hyphal and pseudohyphae formation, Inhibited biofilm formation and filamentation.Downregulated virulence-associated genes including **ERG4, HWP1, and CHT2**.	Evaluated primarily in an *in vitro* oral biofilm model; no *in vivo* validation.
(Takano et al., 2023)	*In vitro*	Japan	*In vitro* adhesion and biofilm model	*Lactobacillus* strains and CFS	*C. albicans*	Adhesion, biofilm assays, hyphal formation assay, MIC	Inhibition of adhesion, biofilm formation, and hyphal development	Inhibited adhesion, hyphal development, and biofilm formation.	Lack of *in vivo* validation and mechanistic analyses.
(do Carmo et al., 2016)	*In vitro*	Brazil	*Reference Lactobacillus strains tested against C. albicans ATCC 18804 and other genital pathogens using HeLa-cell adhesion, mucin binding, co-aggregation, biofilm, and overlay inhibition models*	*L. fermentum* ATCC 23271	*C. albicans*, *C. glabrata*, *C. tropicalis*, *C. krusei*	Adhesion, biofilm assays	HeLa adhesion assay, cell-surface hydrophobicity assay, mucin binding, exclusion/displacement assays, co-aggregation assay, and overlay antimicrobial assay	Strong inhibition of adhesion and biofilm formation.	but all data were generated *in vitro* using HeLa, mucin, and plastic-surface models, with no animal or clinical validation
(Buonanno et al., 2025)	*In vitro* and *in vivo*	Italy	*In vitro epithelial cell model (A-431 vaginal epithelial cells) and Galleria mellonella infection model*	*Kluyveromyces marxianus* and *S. boulardii*	*C. albicans*	Molecular identification, probiotic characterization, antifungal activity, virulence inhibition, and safety assessment	Inhibition of adhesion and biofilm formation, suppression of virulence traits, epithelial protection, probiotic colonization, host immune modulation	Demonstrated strong probiotic potential with significant antifungal activity inhibition of virulence traits, biofilm formation, and adhesion, along with protective effects on epithelial cells and a favourable safety profile.	Findings were primarily derived from laboratory and invertebrate models, limiting direct clinical translation.
(de Barros et al., 2018)	*In vitro* and *in vivo*	Brazil	*Screening of 25 oral clinical Lactobacillus isolates against C. albicans ATCC 18804 and Caenorhabditis elegans coinfection model for in vivo testing.*	*L. paracasei* 28.4	*C. albicans*	*In vitro* filamentation assay, SEM analysis, qRT-PCR of hypha-associated genes, *C. elegans* infection model, survival analysis	Anti-filamentation activity was associated with downregulation of *TEC1* and *UME6* and was not explained by pH reduction alone; secreted probiotic metabolites were implicated but not identified	educed *C. albicans* filamentation and hypha-associated gene expression, improved *C. elegans* survival, inhibited fungal virulence, and demonstrated probiotic potential against candidiasis.	Exact active compounds were not identified; the *in vivo* work relied on a non-mammalian *C. elegans* model; no clinical validation was performed
(Matsubara et al., 2016)	*In vitro*	Brazil	*In vitro biofilm model*	*L. rhamnosus*, *L. casei*, *L. acidophilus*	*C. albicans*	Biofilm assay, CLSM, SEM	Inhibition of early biofilm formation, suppression of yeast-to-hyphal transition, interference with biofilm maturation	Inhibited biofilm maturation and yeast-to-hypha transition.	Limited to *in vitro* observations without mechanistic molecular analysis or *in vivo* validation.
(Rossoni et al., 2020)	*In vitro* and *in vivo*	Brazil	*In vitro planktonic and biofilm models and Galleria mellonella infection model*	*L. paracasei* 28.4 postbiotic (CFS)	*C. auris*	MIC/MFC determination, killing kinetics, biofilm inhibition assays, persister cell susceptibility testing, and *Galleria mellonella* infection model with immune response analysis.	Postbiotic-mediated antifungal activity, biofilm disruption, eradication of persister cells, immune stimulation, host protection	Postbiotic exhibited potent antifungal and anti-biofilm activity against *Candida auris*, eradicated persister cells, enhanced host immune response, improved survival in *Galleria mellonella*, and showed no toxicity.	The study utilized an invertebrate infection model, and the active postbiotic compounds were not completely characterized.

ATCC, American Type Culture Collection; CFS, cell-free supernatant; CLSM, confocal laser scanning microscopy; GC -MS, gas chromatography -mass spectrometry; MIC, minimum inhibitory concentration; MFC, minimum fungicidal concentration; qRT-PCR, quantitative real-time polymerase chain reaction; SEM, scanning electron microscopy; TEM, transmission electron microscopy. Bold text indicates specific virulence-associated genes reported to be downregulated following probiotic treatment.

Most studies employed *in vitro* experimental designs, while several also incorporated *in vivo* models such as murine models and *Galleria mellonella* larvae and *Caenorhabditis elegans*, to evaluate antifungal efficacy and host survival outcomes.

The probiotic strains investigated predominantly belonged to the genus *Lactobacillus*, particularly *L. rhamnosus*, *L. paracasei*, *L. fermentum*, and *L. crispatus*. Additional genera included *Bifidobacterium*, *Streptococcus*, and *Enterococcus*. The predominance of *Lactobacillus* species among the included studies suggest that they represent the most extensively investigated probiotic against *Candida* infections owing to their well-established antifungal properties. Several studies also evaluated probiotic-derived cell-free supernatants and postbiotic formulations. The primary target pathogen studied was *Candida albicans*, although several studies also evaluated NAC including *Candida glabrata*, *Candida tropicalis*, *Candida krusei*, *Candida parapsilosis*, and *Candida famata*. Though current probiotic research remains largely focused on *Candida albicans*, whereas evidence regarding NAC species and emerging multidrug-resistant pathogens such as *Candida auris* remains comparatively limited, highlighting an important gap in the existing literature.

Across studies, probiotics consistently demonstrated antifungal and antibiofilm activity through multiple mechanisms, including inhibition of fungal growth, suppression of germ tube and hyphal formation, disruption of biofilm development, inhibition of adhesion, and downregulation of virulence-associated genes as shown in **Figure 2**.

**Figure 2:**
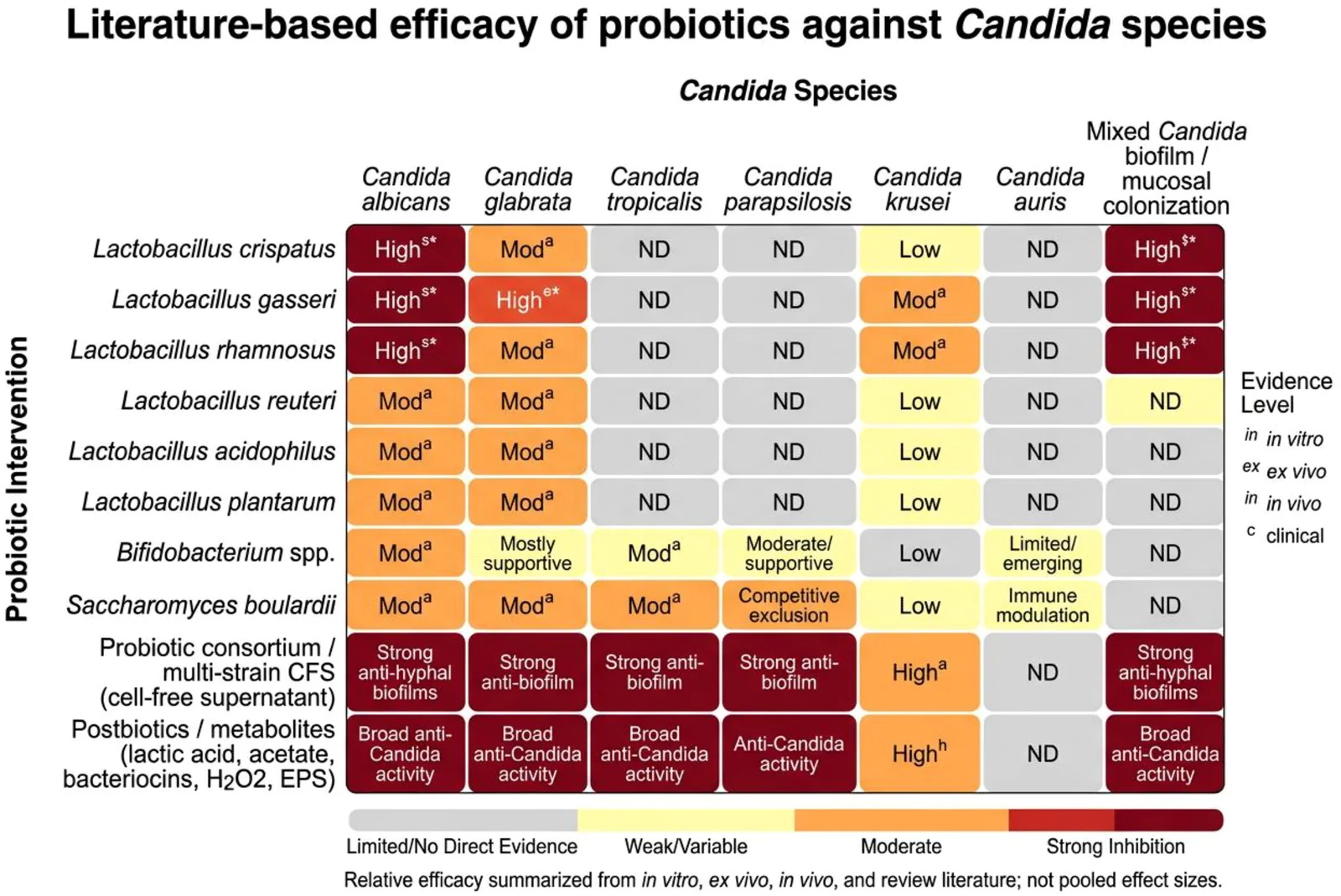
Evidence heat map summarizing probiotic and postbiotic-mediated anti-Candida activities across included studies.

### 3.3 Anti-*Candida* mechanisms of probiotics

The included studies consistently demonstrated that probiotics exert anti-*Candida* effects through multiple complementary mechanisms targeting fungal growth, virulence, biofilm formation, and host-pathogen interactions. The principal mechanisms identified across the included studies were.

- inhibition of *Candida* growth and adhesion
- suppression of yeast-to-hyphal transition
- disruption and prevention of biofilm formation
- downregulation of virulence-associated genes (including *ALS3, HWP1, CPH1, EFG1*, and *ECE1*)
- inhibition of hydrolytic enzyme activity such as proteases and phospholipases
- production of antimicrobial metabolites including organic acids and hydrogen peroxide
- modulation of host immune responses enhancement of antifungal defense and restoration of microbial homeostasis **Figure 3**.

**Figure 3:**
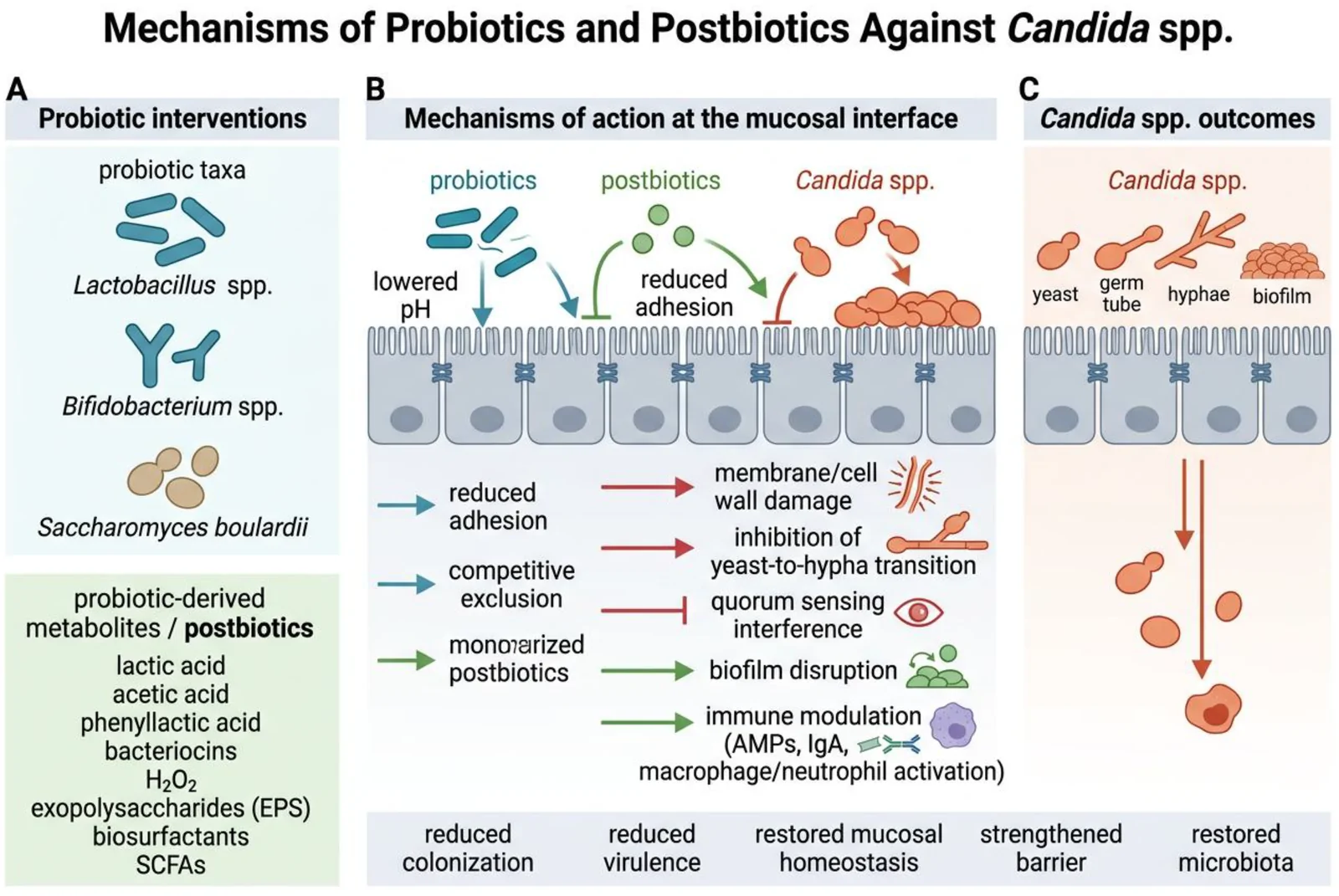
Proposed mechanisms underlying probiotic and postbiotic mediated anti-*Candida* activity. **(A)** Probiotic interventions, including probiotic taxa and probiotic-derived metabolites/postbiotics. **(B)** Mechanisms of action at the mucosal interface, including reduced adhesion and competitive exclusion, membrane, cell wall damage, inhibition of yeast-to-hypha transition, quorum-sensing interference, biofilm disruption, and immune modulation. **(C)** *Candida spp. * outcomes, including reduced colonization, virulence, biofilm formation, and restoration of mucosal homeostasis and microbiota.

Several studies additionally reported immunomodulatory effects, including enhancement of host antifungal defense responses and restoration of microbial homeostasis. These mechanisms were observed against both *C. albicans* and NAC species across *in vitro* and *in vivo* experimental systems.

### 3.4 Quality assessment of included studies

The methodological quality assessment of the included studies is summarized in **Table 2**. Overall, the included studies demonstrated high methodological quality, with total quality scores ranging from 7 to 8 out of a maximum possible score of 8. All studies clearly reported their objectives, study design, probiotic intervention, target *Candida* species, experimental methodology, and primary outcomes. Minor methodological limitations were primarily related to incomplete quantitative outcome assessment and statistical reporting or insufficient description of control/comparator groups in a small number of studies. Despite the overall high methodological quality, considerable heterogeneity remained with respect to probiotic strains, experimental models, treatment conditions, outcome measures, and methodological approaches, which precluded quantitative meta-analysis. No study was classified as moderate or low quality according to the predefined methodological appraisal criteria.

### 3.5 Risk of bias assessment

Most included studies demonstrated a low to moderate risk of bias within each assessed methodological domain. Most studies clearly described the aim of experiments, interventions, outcomes, and methodology properly.

However, several studies demonstrated unclear risk of bias within domains such as comparator standardization, blinding and outcome assessment, and methodological reporting. Differences in experimental design and reporting led to heterogeneity of methodology quality.

Risk-of-bias visualization was performed using the robvis tool to generate traffic-light and summary plots for interpretation of methodological consistency across included studies as shown in **Figure 4**.

**Figure 4:**
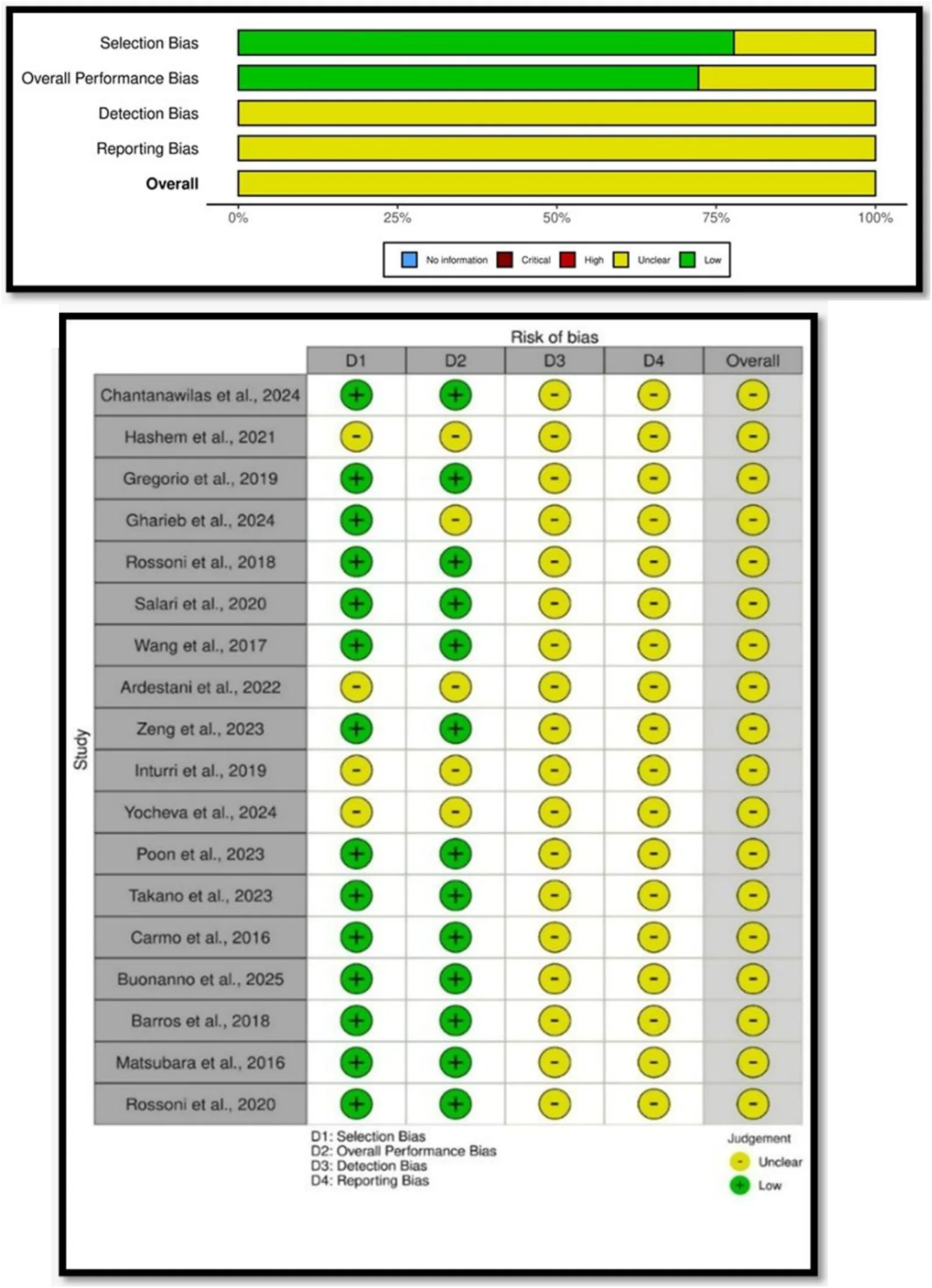
Risk-of-bias summary and graphical representation of included studies. Green indicates low risk of bias, yellow indicates unclear risk of bias, and red indicates high risk of bias.

## 4 Discussion

The incidence of *Candida* species infections has increased significantly over recent decades, with these organisms shifting from commensal microorganisms to opportunistic pathogens capable of causing both mucosal and systemic infections, particularly among immunocompromised populations. This increase is largely driven by widespread antibiotic use, immunosuppressive therapies, invasive medical procedures, and a growing population of susceptible individuals. In parallel, the emergence of antifungal resistance, particularly among *Candida albicans* and non-*albicans Candida* (NAC) species, has further complicated clinical management, highlighting the urgent need for alternative therapeutic strategies (Bongomin et al., 2017; Kim and Kang, 2019).

The findings of this systematic review demonstrate substantial preclinical evidence supporting the antifungal and antibiofilm potential of probiotics against *Candida* species. Across the included studies, probiotic strains, predominantly belonging to *Lactobacillus, Bifidobacterium, and Saccharomyces* species, demonstrated broad antifungal activity through multiple complementary mechanisms (Wang et al., 2017; Kang et al., 2018; Carvalho et al., 2021). These effects included inhibition of fungal proliferation, suppression of yeast-to-hyphal transition, disruption of biofilm formation, and modulation of host immune responses, collectively contributing to reduced *Candida* colonization and pathogenicity. These observations are consistent with emerging evidence highlighting probiotics as microbiome-based therapeutic *Candida*tes capable of modulating fungal colonization, restoring microbial homeostasis, and enhancing host defense responses against opportunistic fungal pathogens (Hu et al., 2017; Wu et al., 2022; Divyashree et al., 2023).

One of the most important findings of this review was the consistent inhibition of *Candida* biofilm formation across multiple experimental models. Biofilms represent major virulence determinants associated with reduced antifungal susceptibility, chronic infection, and persistence on mucosal surfaces and medical devices. Several included studies demonstrated that probiotics inhibited biofilm biomass, reduced biofilm maturation, and modulated expression of virulence-associated genes, including ALS3, HWP1, CPH1, EFG1, and ECE1. These findings are particularly significant because biofilm-associated *Candida* cells exhibit reduced susceptibility to conventional antifungal agents (Rossoni et al., 2018b; Hefzy et al., 2021). Therefore, targeting biofilm development represents an important strategy for reducing *Candida* pathogenicity and overcoming antifungal resistance (Cavalheiro and Teixeira, 2018; Ajetunmobi et al., 2023).

Despite consistent evidence supporting probiotic-mediated anti-*Candida* activity, the magnitude of antifungal effects varied considerably among studies, highlighting the importance of strain-specific characteristics. Although *Lactobacillus* species represent the most extensively investigated probiotic group against *Candida* infections, antifungal activity is not uniform among individual strains. Wang et al. demonstrated strong inhibition of *C. albicans* growth and yeast-to-hyphal transition by *Lactobacillus crispatus*, associated with suppression of virulence-related gene expression (Wang et al., 2017). In contrast, Hashem et al. reported pronounced antibiofilm activity with *Lactobacillus acidophilus and Lactobacillus plantarum* cell-free supernatants against clinical *Candida* isolates (Hashem and Hashem, 2021). Similarly, Rossoni et al. demonstrated that *Lactobacillus paracasei* strains reduced biofilm formation and modulated *Candida* virulence-associated gene expression (Rossoni et al., 2018a). These differences suggest that probiotic efficacy depends not only on probiotic genus but also on strain-specific properties, including metabolite production, adhesion capacity, ecological origin, surface characteristics, and interaction with *Candida* cells (Vazquez-Munoz and Dongari-Bagtzoglou, 2021; Campos et al., 2026). Moreover, variations in experimental design, including differences in Candida isolates, growth conditions, probiotic concentration, exposure duration, and outcome assessment methods, may contribute to the variability observed among studies and should be considered when interpreting comparative antifungal efficacy.

Differences in susceptibility between *C. albicans* and NAC species further indicate that probiotic activity may be species dependent. Although several studies demonstrated activity against NAC species, including *C. glabrata, C. tropicalis, C. krusei, and C. parapsilosis*, most mechanistic investigations continue to focus on *C. albicans*. Inturri et al. and Salari et al. reported inhibitory effects of probiotic-derived products against multiple *Candida* species, suggesting broader antifungal potential beyond *C. albicans* (Inturri et al., 2019; Salari and Ghasemi Nejad Almani, 2020). However, limited information is available regarding whether probiotic-mediated mechanisms are conserved across *Candida* species or whether species-specific strategies are required. This limitation is particularly relevant for emerging multidrug-resistant pathogens such as *Candida auris.* Rossoni et al. demonstrated that *Lactobacillus paracasei*-derived postbiotics exhibited activity against *C. auris* by disrupting biofilms, reducing persister cells, and improving survival in an experimental infection model (Rossoni et al., 2020). Nevertheless, additional studies are required to determine whether these promising findings can be translated into clinically effective interventions.

The antifungal effects of probiotics appear to result from the combined action of multiple complementary mechanisms rather than a single inhibitory pathway. Organic acids, hydrogen peroxide, bacteriocin-like compounds, and other bioactive metabolites modify the local microenvironment, thereby reducing fungal adhesion, inhibiting yeast-to-hyphal transition, and preventing biofilm maturation (Vazquez-Munoz and Dongari-Bagtzoglou, 2021; Campos et al., 2026). In addition, probiotics may enhance colonization resistance by restoring microbial homeostasis, strengthening epithelial barrier integrity, and modulating innate and adaptive immune responses. The simultaneous targeting of multiple pathogenic pathways may explain the broad anti-*Candida* activity observed across different experimental models and probiotic strains (Wu et al., 2022; Divyashree et al., 2023).

An important distinction among the included studies was the use of live probiotic organisms compared with probiotic-derived metabolites and postbiotic formulations. Studies evaluating live probiotic strains primarily emphasized mechanisms such as competitive exclusion, adhesion inhibition, and restoration of microbial balance. In contrast, studies investigating cell-free supernatants and postbiotics demonstrated direct antifungal activity mediated by secreted metabolites. Rossoni et al. showed that Lactobacillus paracasei-derived postbiotics exhibited potent activity against *C. auris*, including biofilm disruption, reduction of persister cells, and enhancement of host protection in an experimental infection model (Rossoni et al., 2020). Similarly, several studies demonstrated antifungal effects of probiotic-derived supernatants through organic acids and antimicrobial compounds (Inturri et al., 2019; Hefzy et al., 2021). These findings suggest that postbiotics may provide advantages related to formulation stability and defined antimicrobial activity; however, the active compounds responsible for these effects remain incompletely characterized.

Previous studies have demonstrated the potential role of probiotics in fungal infections, particularly against *C. albicans*, emphasizing their antimicrobial and immunomodulatory properties. However, most earlier reviews focused on selected probiotic strains, clinical applications, or specific aspects of *Candida* pathogenesis (Ribeiro et al., 2020). For example, previous systematic reviews evaluated the role of probiotics in reducing oral *Candida* colonization and prevention of candidiasis but provided limited discussion regarding strain-level differences, biofilm inhibition, virulence regulation, and activity against emerging *Candida* species (Hu et al., 2019; Mundula et al., 2019). In contrast, the present systematic review provides a broader synthesis of experimental and mechanistic evidence by integrating findings involving *C. albicans*, NAC species, and the emerging multidrug-resistant pathogen *C. auris.* Furthermore, this review highlights multiple anti-*Candida* mechanisms, including fungal growth inhibition, virulence suppression, biofilm disruption, immune modulation, and the therapeutic potential of probiotic-derived metabolites and postbiotics.

Emerging evidence further suggests that probiotics may influence Candida pathogenesis through additional mechanisms, including quorum-sensing interference, production of bacteriocins and antimicrobial peptides, release of extracellular vesicles, enhancement of epithelial barrier integrity, and restoration of microbiota homeostasis (Conti and Gaffen, 2015; Bron et al., 2017; Lebeer et al., 2018; Krzyźek et al., 2023; Tukaram et al., 2025; Debta et al., 2026). Probiotic-derived effector molecules and secreted vesicles may facilitate host–microbe communication and contribute to antifungal activity by modulating fungal growth and host responses. Furthermore, probiotics can influence innate and adaptive immune pathways, including Toll-like receptor (TLR)-mediated signalling, NF-κB activation, and IL-17-dependent antifungal immunity, which are critical for mucosal defense against Candida albicans (Conti and Gaffen, 2015; Bron et al., 2017). These complementary mechanisms further support the concept that probiotics exert multifactorial rather than single-target antifungal effects.

Despite these promising findings, several limitations should be considered. Most available evidence was derived from *in vitro* experiments and small-scale *in vivo* models, which may not fully reproduce the complex interactions between probiotics, *Candida*, host immunity, and resident microbiota occurring during human infection. Considerable heterogeneity was observed among studies regarding probiotic strains, dosages, formulations, experimental models, treatment duration, and outcome assessment methods, limiting direct comparison between studies. Due to this methodological heterogeneity, quantitative meta-analysis was not considered appropriate. In addition, variability in outcome reporting, limited statistical analysis, and incomplete characterization of probiotic-derived compounds in some studies contributed to uncertainty regarding the strength of available evidence.

Translation of probiotic-based strategies into clinical practice requires consideration of several practical and regulatory challenges. The effectiveness of probiotic interventions may depend on formulation stability, microbial viability, dosage optimization, and delivery approaches. Furthermore, regulatory frameworks for probiotic-based therapeutics vary between countries, creating challenges for clinical approval and implementation. Patient safety must also be carefully considered, particularly among immunocompromised populations, where rare cases of probiotic-associated infections have been reported. Therefore, standardized formulations, rigorous safety evaluations, and adequately powered clinical trials are essential before probiotics can be routinely incorporated into candidiasis management.

Nevertheless, the consistency of antifungal and antibiofilm effects observed across diverse experimental systems provides a strong rationale for further investigation. Future research should focus on standardized clinical trials evaluating strain-specific efficacy, optimal dosing strategies, safety profiles, and long-term outcomes. In addition, mechanistic studies investigating probiotic–host–fungal interactions and metabolite profiling may provide deeper insights into antifungal pathways. Emerging therapeutic approaches involving synbiotics, postbiotics, and targeted microbiome modulation may further improve the stability, specificity, and therapeutic applicability of probiotic-based strategies for candidiasis prevention and management.

## 5 Conclusion

This systematic review highlights substantial preclinical evidence supporting the antifungal, antibiofilm, and immunomodulatory effects of probiotics against *Candida species*, *including Candida albicans, non-albicans Candida species, and Candida auris.* Probiotic strains, especially *Lactobacillus*, consistently demonstrated inhibitory effects on fungal growth, adhesion, yeast-to-hyphal transition, biofilm formation, and virulence-associated pathways across diverse experimental models.

However, these findings are derived predominantly from heterogeneous preclinical studies, and their direct translation to clinical practice remains uncertain. Variations in probiotic strains, experimental models, methodologies, and outcome measures limit the generalizability of the current evidence. Therefore, although probiotics represent promising microbiome-based therapeutic candidates, their clinical efficacy, safety, optimal dosing strategies, and long-term outcomes remain to be established through well-designed clinical studies.

Future research should prioritize standardized mechanistic investigations and well-designed clinical trials to determine strain-specific efficacy, optimal therapeutic formulations, dosing strategies, long-term safety, and clinical applicability of probiotics and postbiotics in candidiasis prevention and management.

## Data Availability

The original contributions presented in the study are included in the article/**Supplementary Material**. Further inquiries can be directed to the corresponding author.
