# Supplementary material for: Harnessing probiotics against candidiasis: a systematic review of experimental and mechanistic evidence
Source: Front Cell Infect Microbiol. 2026 Sep 24;16:1894309. doi: 10.3389/fcimb.2026.1894309 (PMC13645589; doi:10.3389/fcimb.2026.1894309)
Supplement: Supplementary file 1 [file Table1.docx]

| **Database** | **Search strategy** |
| --- | --- |
| PubMed | ("probiotic"[Title/Abstract] OR "Lactobacillus"[Title/Abstract] OR "Bifidobacterium"[Title/Abstract] OR "Saccharomyces"[Title/Abstract]) AND ("Candida"[Title/Abstract] OR "candidiasis"[Title/Abstract]) |
| Scopus | TITLE-ABS-KEY ("probiotic" OR "Lactobacillus" OR "Bifidobacterium" OR "Saccharomyces") AND TITLE-ABS-KEY ("Candida" OR "candidiasis") |
| Web of Science | TS=("probiotic" OR "Lactobacillus" OR "Bifidobacterium" OR "Saccharomyces") AND TS=("Candida" OR "candidiasis") |

**Supplementary Table S1. Database-specific search strategies**
